# Systemic lupus erythematosus: Auditing standard of care in Qatar

**DOI:** 10.5339/qmj.2026.9

**Published:** 2026-03-17

**Authors:** Mohamad Safieh, Abdul-Wahab Al-Allaf

**Affiliations:** 1 Internal Medicine, Hamad General Hospital, Doha, Qatar; 2 Rheumatology, Hamad General Hospital, Doha, Qatar *Email: msafieh@hamad.qa

**Keywords:** Systemic lupus erythematosus, SLE, clinical audit, quality indicators, hydroxychloroquine, Qatar

## Abstract

**Background:**

Systemic lupus erythematosus (SLE) is a complex autoimmune disease with heterogeneous clinical presentations influenced by ethnicity, geography, and healthcare practices. This audit evaluated adherence to key quality indicators for SLE management at Hamad General Hospital (HGH), a major public tertiary care center in Doha, Qatar, in line with international standards.

**Methods:**

A retrospective cross-sectional audit was conducted between March and July 2024 involving 61 patients with SLE attending the rheumatology clinic at HGH. This audit assessed adherence to four selected key quality indicators: hydroxychloroquine (HCQ) use, annual proteinuria screening, annual ophthalmologic screening for HCQ-related retinopathy, and medication reconciliation documentation during the most recent clinic visit.

**Results:**

All patients were initiated on HCQ, with 11.5% discontinuing it due to adverse effects. Annual proteinuria screening was completed in 95% of patients. Ophthalmologic screening for HCQ-related retinopathy was documented in 65%, and medication reconciliation was completed in 72% of cases. The cohort represented a total of 21 nationalities, including Qataris who accounted for 31%–a proportion higher than their estimated 11.6% representation in Qatar’s national population.

**Conclusion:**

While adherence to HCQ initiation and renal monitoring met international benchmarks, ophthalmologic screening and medication reconciliation rates were suboptimal and required improvement. Targeted system-level and educational interventions are planned to enhance adherence to these standards. Re-audit following implementation will be essential to assess progress and to sustain the quality of care.

## 1. INTRODUCTION

Systemic lupus erythematosus (SLE) is a chronic, multisystem autoimmune disease characterized by diverse clinical and laboratory manifestations.^B1^ Studies from North America, Europe, and the Arab world have highlighted the heterogeneity of SLE, which is influenced by genetic, ethnic, and environmental factors, leading to variations in disease phenotype and severity among different populations.^B2^^–^^B6^ Additionally, race was associated with differences in the severity of the disease and associated mortality.^B7^ In the Arab Gulf region, including Saudi Arabia, Oman, and the United Arab Emirates, research underscored these racial and ethnic differences in SLE presentation and outcomes.^B8^^–^^B10^

This audit aims to evaluate adherence to internationally recognized key performance indicators (KPIs) for SLE management at Hamad General Hospital (HGH), a major public hospital in Doha, Qatar, based on the European Alliance of Associations for Rheumatology (EULAR) and the American College of Rheumatology (ACR) guidelines. The selected KPIs include hydroxychloroquine (HCQ) use in all eligible patients, annual screening for proteinuria, annual screening for HCQ-related retinopathy, and documentation of medication reconciliation at the most recent visit. The current audit also showed significant clinical variability among the multiethnic SLE patient population in Qatar.

## 2. METHODS

A retrospective cross-sectional audit was conducted between March and July 2024 involving 61 patients with SLE, all of whom attended the rheumatology outpatient clinic at HGH, a public tertiary care facility in Doha, Qatar. This audit was conducted as part of the rheumatology department’s quality improvement activities and, in line with local regulations, did not require formal approval from the institutional review board and the medical research center.

Clinical and demographic data were extracted from the electronic medical record system, including age, sex, nationality, clinical manifestations, and treatment regimens. Compliance with the following quality indicators was assessed: HCQ use—percentage of patients initiated on HCQ unless contraindicated; annual ophthalmology screening—for HCQ-related retinopathy; annual proteinuria screening—via urinalysis or urine protein-to-creatinine ratio; and medication reconciliation—conducted electronically at the most recent visit. Medication reconciliation was validated only when documented through the designated electronic workflow in the hospital’s standard electronic medical record system, not through free-text documentation.

The EULAR and the ACR guidelines were used as a benchmark for the selected quality indicators, including the HCQ use for all SLE patients unless contraindicated, annual screening for lupus nephritis, and ophthalmic screening for patients on HCQ.^B11^,^B12^

## 3. RESULTS

The audit included 61 patients, of whom 94% were female (female-to-male ratio, 7.7:1). The mean age was 43.3 years. Qataris represented 31% of the cohort, a proportion higher than their approximately 12% representation in Qatar’s national population, while the remaining 42 patients represented 20 additional different nationalities (Table 1).

**Figure 1:**
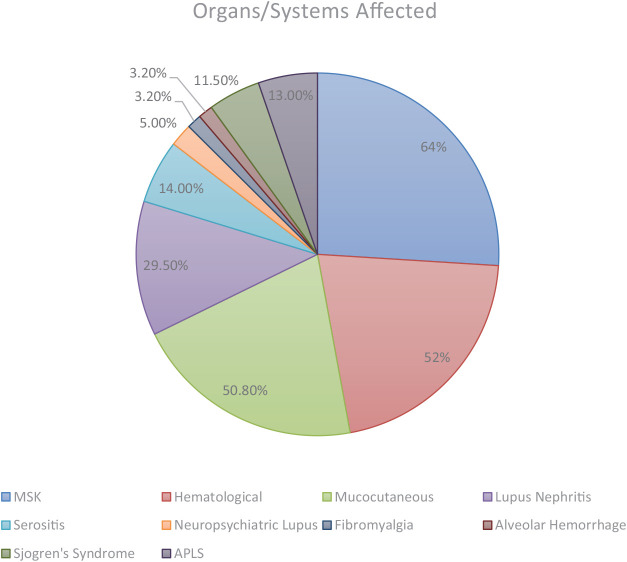
Distribution of organ system involvement among patients with systemic lupus erythematosus in the study cohort.

The most frequently observed clinical manifestations were musculoskeletal involvement (64%), hematological abnormalities (52.4%), mucocutaneous manifestations, including Raynaud phenomenon (50.8%), and lupus nephritis (29.5%). Less common findings included serositis (14.7%), neuropsychiatric lupus (5%), fibromyalgia (3.2%), pulmonary hemorrhage (3.2%), Sjögren’s syndrome (11.5%), and antiphospholipid syndrome (13%); (Table 2; Figure 1).

HCQ was initiated in 100% of patients; however, 11.5% discontinued the drug due to adverse effects. Other commonly used agents included mycophenolic acid (26.2%), azathioprine (24.5%), tacrolimus (6.5%), rituximab (6.5%), and belimumab (5%); (Table 3).

**Figure 2:**
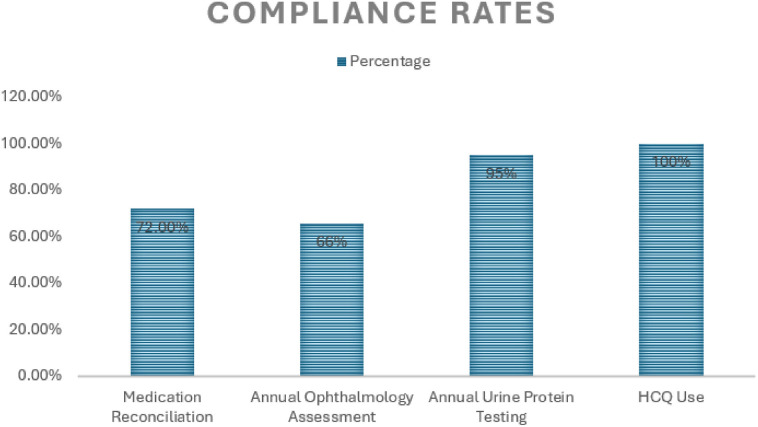
Compliance rates with selected quality indicators for systemic lupus erythematosus management at the Rheumatology Clinic, Hamad General Hospital, Qatar.

Compliance with the predefined key quality indicators assessed in this audit is comprehensively detailed in Table 4 below and Figure 2.

## 4. DISCUSSION

This audit highlights the ethnic and clinical diversity of SLE patients in Qatar, reflecting the country’s multinational profile. Despite the modest sample size, our findings affirm the heterogeneous nature of SLE, consistent with prior studies. Qataris accounted for 31% of our sample—more than double their estimated 11.6% representation in Qatar’s total population.^B13^ This overrepresentation might reflect that nationals have greater familiarity with the healthcare system compared to expatriate groups. An earlier population-based study conducted during 2006 to 2010 reported statistically significantly higher inpatient and outpatient service utilization among Qatari nationals compared with non-nationals.^B14^ Given the substantial population growth and healthcare system expansion in Qatar since that period, these findings should be interpreted cautiously and may not fully reflect current utilization patterns, though.

### 4.1 Review of quality indicators

**4.1.1 HCQ use for all SLE patients**

Universal initiation of HCQ suggests strong adherence to EULAR/ACR recommendations.^B11^^,^^B12^ The 11.5% discontinuation rate was attributable to documented adverse effects or patient-reported inefficacy. One patient discontinued HCQ due to intolerable dizziness and blurred vision. Other Adverse events leading to discontinuation included allergic reaction, pruritus with skin pigmentation and dryness, HCQ-associated retinopathy, and intolerable nausea. In addition, two patients stopped the drug because they perceived it as ineffective.

**4.1.2 Annual proteinuria screening**

Achieving 95% compliance aligns with EULAR/ACR recommendations for early detection and management of lupus nephritis.^B11^^,^^B12^

**4.1.3 Ophthalmologic screening for HCQ retinopathy**

At 65%, this falls short of the American Academy of Ophthalmology (AAO) recommendations. The latest AAO guidelines recommend HCQ retinopathy screening during the first year of HCQ use for all patients, regardless of risk, and yearly screening after 5 years of continuous use of HCQ medication.^B15^ Contributing factors may include poor patient follow-up, missed referrals, and non-captured external assessments. Our rate, while better than regional benchmarks (e.g., a 2024 Saudi study reporting 36.5% compliance),^B16^ still requires improvement.

**4.1.4 Medication reconciliation**

With 72% compliance, performance remains below our institutional target of 90%. Factors include physician rotation, lack of uniform training in the electronic patient record system, and reliance on narrative documentation rather than structured entries.

### 4.2 Recommendations and quality improvement plan

We propose implementing standardized referral protocols between rheumatology and ophthalmology, creating Electronic Medical Records (EMR) alerts for overdue screenings, and strengthening patient education on the importance of regular eye examinations. For medication reconciliation, targeted training sessions for clinicians, integration of reconciliation checklists into EMR templates, and periodic audit-feedback cycles are recommended.

### 4.3 Audit strengths and limitations

This audit was conducted at one of Qatar’s largest tertiary care centers, providing access to a diverse patient population and a wide spectrum of SLE phenotypes. It offers valuable insights into real-world practice patterns and adherence to guidelines within a multicultural healthcare setting. The main limitation is the relatively small sample size, which may affect the generalizability of findings. Future audits with larger cohorts, particularly focusing on Qatari nationals, could provide more robust epidemiological and clinical insights.

## 5. CONCLUSION

This audit underscores the significant ethnic diversity and clinical heterogeneity among SLE patients receiving care at HGH in Qatar. While adherence to international recommendations for HCQ initiation and renal monitoring was high, ophthalmologic screening for HCQ-related retinopathy and medication reconciliation fell short of international benchmarks. Addressing these issues is important because it will enhance early detection of treatment-related toxicity, improve medication safety, and optimize long-term outcomes for SLE patients. This will require targeted system-level interventions, such as standardized referral protocols, EMR-based alerts, and clinician education programs, alongside patient engagement strategies to improve follow-up adherence. We plan to revisit these indicators in one year to evaluate the impact of the recommended changes and to guide further improvements in care.

## CONFLICT OF INTEREST

The authors declare no conflicts of interest.

## INFORMED CONSENT

Informed consent is not required for this study.

## AUTHOR CONTRIBUTION

MS and AA-A conceptualized the study and contributed to the development of the study design. MS collected and curated the data, participated in the interpretation and discussion of the findings, and drafted the initial manuscript. AA-A provided methodological oversight, contributed to the interpretation and discussion of the results, critically revised the manuscript for important intellectual content, and supervised the overall conduct of the project. Both authors reviewed and approved the final manuscript

## Figures and Tables

**Table 1.** Baseline demographic characteristics of patients with systemic lupus erythematosus attending Hamad General Hospital, Qatar, between March and July 2024.

Total number of patients	*N* = 61	Percent (%)
Gender		
Female	57	94%
Male	4	6%
Female-to-male ratio	7.7:1	
Age, years (mean ± SD)	43.3 ± 10.6 years	
Nationality	21	
Qatari	19	31%
Other 20 nationalities	42	69%
Filipino*		14.7%
Egyptian*		9.8%
Jordanian*		8.2%

*Most common nationalities. Remaining nationalities: Yemeni, Tunisian, Emirati, Indian, Bangladeshi, Canadian, Iranian, Bahraini, Sri Lankan, Sudanese, Iraqi, Lebanese, American, Syrian, Algerian, Australian, and Chadian).

**Table 2.** Clinical manifestations among patients with systemic lupus erythematosus in the study sample.

Clinical feature	Prevalence
Musculoskeletal (arthritis/arthralgia)	64% (*n* = 39)
Hematological	52.4% (*n* = 32)
Anemia	16.4%
Neutropenia	8.2%
Thrombocytopenia	6.5%
Bicytopenia	3.3%
Pancytopenia	5%
Antiphospholipid syndrome	13%
Mucocutaneous (including Raynaud)	50.8% (*n* = 31)
Lupus nephritis	29.5% (*n* = 18)
Serositis (pericarditis/pleuritis)	14.7% (*n* = 9)
Neuropsychiatric lupus	5% (*n* = 3)
Fibromyalgia	3.2% (*n* = 2)
Pulmonary/alveolar hemorrhage	3.2% (*n* = 2)
Sjogren’s syndrome	11.5% (*n* = 7)
Antiphospholipid syndrome	13% (*n* = 8)

**Table 3.** Pharmacologic management of systemic lupus erythematosus in the study cohort.

Drug	Prevalence
Hydroxychloroquine (HCQ)	100% (*n* = 61)
Mycophenolic acid (MMF)	26.2% (*n* = 16)
Azathioprine	24.5% (*n* = 15)
Tacrolimus	6.5% (*n* = 4)
Rituximab	6.5% (*n* = 4)
Belimumab	5% (*n* = 3)
Methotrexate	1.6% (*n* = 1)
Cyclosporine	1.6% (*n* = 1)

**Table 4.** Compliance with key quality indicators for systemic lupus erythematosus management among patients attending Hamad General Hospital Rheumatology Clinic, Qatar, during the audit period (March–July 2024).

Measure	Compliance/prevalence
Medication reconciliation	72% (*n* = 44)
Annual ophthalmology referral	65.5% (*n* = 40)
Annual urine protein testing	95% (*n* = 58)
Hydroxychloroquine initiation	100% (*n* = 61)
Continuation	88.5% (*n* = 54)
Stopped due to side effects	11.5% (*n* = 7)
